# Controlling the Mixing Performance of Passive Micromixers with Variable Section Units

**DOI:** 10.3390/mi17091082

**Published:** 2026-09-15

**Authors:** Lijun Yang, Yu Hang, Renjie Liu, Qilin Jiang, Zongan Li, Jun Hong, Ye Wu

**Affiliations:** 1 School of Mechanical Engineering, Nanjing Institute of Technology, Nanjing 211167, China; yanglj@njit.edu.cn (L.Y.); fulisayang@163.com (R.L.); jiangqilin@njit.edu.cn (Q.J.); 2 School of Artificial Intelligence, Nanjing Normal University of Special Education, Nanjing 210038, China; 3 School of Electrical and Automation Engineering, Nanjing Normal University, Nanjing 210046, China; 4 School of Electrical and Information Engineering, Hunan Institute of Technology, Hengyang 421002, China; 2013001763@hnit.edu.cn (J.H.); chemwuye@hnit.edu.cn (Y.W.)

**Keywords:** micromixers, mixing efficiency, geometric parameters, variable section units

## Abstract

Changing the structures of microchannels has become an effective strategy for improving the mixing performance of passive micromixers. In this study, we propose a novel micromixer with variable section units through changing the geometric parameters including the type, the position, the number, and the ratio of the variable section units. The effects of these geometric parameters on the mixing performance were numerically investigated and parametrically compared. The results showed that the type of Gra channel, the initial position, a number of 4, and the ratio of 1:4 should be selected. With this selected configuration, the mixing efficiency of the micromixer can be significantly enhanced, with the mixing index exceeding 0.96 at a Reynolds number of 100. These findings demonstrate an effective design strategy for developing passive micromixers.

## 1. Introduction

Micromixers have been widely applied in various fields, including disease diagnosis [1,2], drug synthesis [3], material preparation [4,5], and environmental protection [6], owing to their advantages of low sample consumption, short processing time, and high mixing efficiency. Micromixers can generally be classified into two categories—active and passive mixers—depending on whether external energy is required during the mixing process [7]. Active mixers generate disturbances in the fluid by applying external energy, which disrupts the original streamlines and increases the interfacial contact area between fluids, thereby enhancing mixing efficiency [8,9,10]. However, the use of external energy requires additional driving devices, making the mixing process more complex and costly. Moreover, the applied external energy may adversely affect the activity of biological samples and interfere with chemical reactions.

In contrast, passive mixers utilize specially designed channel structures to manipulate the flow field and generate chaotic advection without requiring external energy. Consequently, passive micromixers offer the advantages of low energy consumption, simple operation, and relatively low cost. To improve their mixing performance, numerous studies have investigated different strategies for optimizing the structural characteristics of passive micromixers. For example, researchers have modified the channel path geometry [11,12,13], altered the inlet configurations [14], designed fractal tree-like structures [15,16], and developed split-and-recombine structures [17,18,19]. In addition, obstacles or baffles have been incorporated into microchannels to manipulate the flow field and enhance mixing [20,21,22]. Usefian et al. investigated the mixing performance of a convergent-divergent micromixer through changing the inlet velocity, number of mixing cycles, and blockage ratios. The results revealed that the mixing efficiency increased with injection velocity, number of cycles, and blockage ratio. In addition, the micromixer with a blockage ratio of 0.25 reached a mixing efficiency of 100% [12]. Rahbarshahlan et al. designed six different inlet geometries and found that the mixing performance was strongly influenced by the location, magnitude, and direction of rotation of vortices, as well as the number of vortices and the Reynolds number. Their results showed that the third geometry, in which one inlet was divided into two equal parts and the two inlets were arranged in non-aligned and parallel positions on opposite sides, exhibited the best overall performance, owing to its high mixing intensity, low mixing cost, and simple fabrication [14]. Aghasi et al. designed six novel micromixers by varying the number and arrangement of mixing units based on the split-and-recombine mechanism. A mixing index higher than 0.92 was achieved for the micromixer with seven alternating mixing units, while the figure of merit increased to 32.5 for the micromixer with five alternating mixing units [19]. Hasanah et al. numerically investigated the mixing performance and flow mechanisms of passive micromixers incorporating Koch fractal obstacle arrays with four different geometries. Their results demonstrated that Koch fractal obstacles enhanced mixing through flow splitting, stretching, and chaotic advection. Among the investigated configurations, the different-side rounded tertiary snowflake fractal exhibited the most stable and highest mixing performance [21].

Among the various structural strategies, passive micromixers with variable section microchannels have attracted considerable attention. This may be due to the fact that the geometric dimensions of variable section vary along the path, which is beneficial for breaking the laminar boundary layer as well as inducing the generation of secondary flows or vortices.

There have been many research projects on passive mixers through changing the sections of the channels. These approaches include the introduction of periodic expansion units [23,24], change of the channel section size [25,26,27], incorporation of fractal structures [28,29,30], and addition of twisted structures [31,32]. Natsuhara et al. developed a planar asymmetric contraction and expansion micromixer with asymmetric vertical obstacle structures. The flow velocities and directions of the two fluids changed differently while alternately crossing the longitudinal centerline of the microchannel. This behavior increased the interfacial contact area between the two fluids and promoted effective mixing [25]. Chen et al. designed variable section micromixers based on the Cantor fractal principle and investigated the effects of fractal structures on mixing efficiency. They found that the mixing efficiency increased with increasing fractal level and channel height, as well as with decreasing spacing between adjacent fractal structures [30]. Najafpour et al. proposed an innovative micromixer incorporating twisted structures and investigated the effects of variations in the twisted geometry on mixing performance. The numerical results demonstrated that the mixing performance was significantly improved in microchannels with twisted patterns because of the curvature-induced secondary flows [31].

Passive micromixers with variable section microchannels offer several advantages, including low energy consumption, high mixing efficiency, low cost, and flexible structural design. Therefore, further investigation into the effects of geometric parameters of variable section channels on mixing performance is warranted. In a previous work [33], we researched the effects of geometric parameters including the channel diameter, channel shape, channel contraction ratio and expansion ratio, and number of the expansion units on the mixing efficiency. The channel was a T-shaped channel with several expansion units. The inlet boundary type of the channel was set as a velocity inlet, and the outlet boundary type of the channel was set as a pressure outlet with the gauge pressure of 0. The flow conditions at both inlets were laminar, and there was no slippage between the fluids and the walls. The parametric comparison results showed that the diameter of 0.2 mm, shape of Sem channel, expansion ratio of 1:3, and number of expansion units of 7 were selected. However, the sections of the expansion units were suddenly changed, demonstrating poorer performance than the gradually changed units at higher Reynolds numbers, as discussed in Section 3.1. In this work, a novel passive micromixer is introduced, with variable section units which were gradually changed. Then, the effects of the geometric parameters such as the type, the position, the number, and the ratio of the variable section units on the mixing performance were explored through the 3D numerical simulation. In the next section, the key parameters set within the simulation are discussed. In the third section, the simulation results of variable section units are shown and discussed. In the fourth section, conclusions are given. This study is expected to provide a new scheme for making passive micromixers for experiments.

## 2. Materials and Methods

### 2.1. Geometric Model

**Figure 1:**
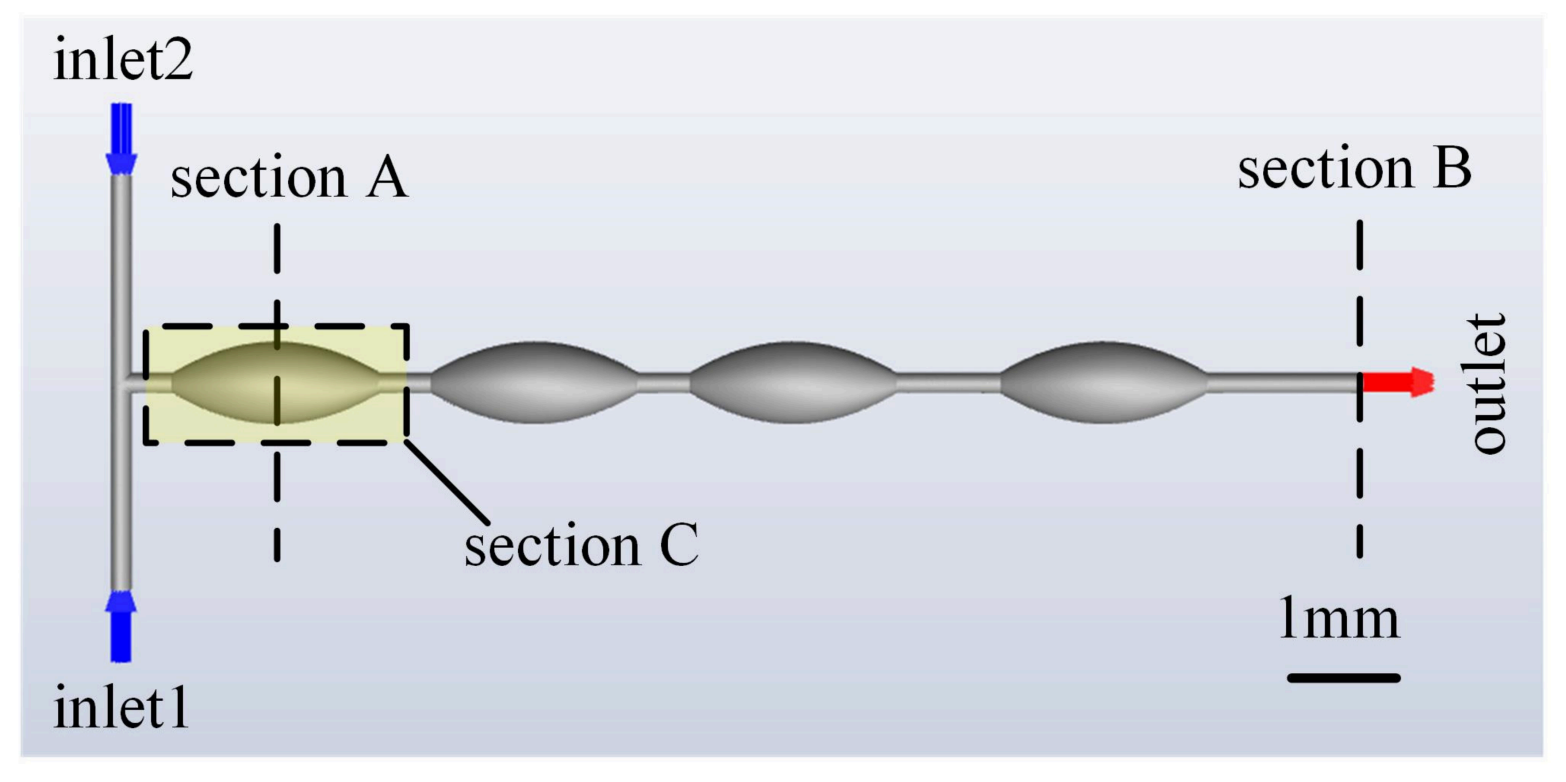
Geometric model of the micromixer.

The basic geometric model of the micromixer consisted of a T-shaped channel incorporating four variable section units. The diameter of the main channel and the maximum diameter of the variable section units were 0.2 mm and 0.6 mm, respectively (see Figure 1). The distance between the two inlets was 4 mm, while the total length of the mixing region was 12 mm. The length of each variable section unit and the distance between the two neighboring units were 2 mm and 0.5 mm. The section at the maximum diameter of the variable section unit was named section A. The section at the outlet was named section B. In addition, the longitudinal section of the variable section unit was named section C.

### 2.2. Governing Equations and Boundary Conditions

The governing equations were established based on the assumptions that the fluid in the micromixer was a continuous, incompressible, Newtonian fluid and that the flow was steady and laminar. Accordingly, the continuity equation, momentum equation, and convection–diffusion equation are given as follows:

(1) ∇⋅U=0

(2) ∂U∂t+U⋅∇U=−∇P+1Re∇2U

(3) $$\frac{\partial C}{\partial t}+U\cdot \nabla C=\frac{1}{Pe}\nabla^{2}C$$

(4) $$Re=\frac{\rho UL}{\mu }$$

(5) $$Pe=\frac{UL}{D}$$

where *U* is the velocity, *P* is the pressure, *Re* is the Reynolds number, *C* is the concentration, *Pe* is the Peclet number, *ρ* is the density of the fluid, *L* is the diameter of the main channel, *μ* is the viscosity, and *D* is the diffusion coefficient.

In the numerical simulation, water and salt water were selected as the working materials with the diffusion coefficient of 1.33 × 10^−9^ m^2^/s, and densities of the two materials were 998.2 kg/m^3^ and 1024 kg/m^3^, respectively. The concentration of the salt water was 3.5%. The viscosities of water and salt water were 1.003 mPa·s and 0.96 mPa·s, respectively. The characteristic length was set to 0.2 mm. The inlet velocity varied from 0.0004 to 2 m/s, corresponding to a Reynolds number range of 0.08–400, as listed in Table 1. A prescribed velocity was imposed at the channel inlets, while the outlet was assigned a zero-gauge-pressure boundary condition. A no-slip boundary condition was applied to all channel walls.

### 2.3. Evaluation Method

The mixing index (*MI*) was used to quantitatively evaluate the mixing performance of the fluids in the micromixer, as defined by Equation (6). In this equation, (*N*) denotes the total number of points on the section being counted, (*C_i_*) represents the concentration or mass fraction of each point on the section being counted, and (*C_m_*) is the expected concentration or mass fraction on the section, which was set to 0.5. The MI could reflect the degree of mixing at any section of the mixing channel and ranged from 0 to 1. In this study, the fluids were considered to be completely mixed when the *MI* exceeded 0.95.

(6) $$MI=1-\sqrt{\frac{\sum {\left(C_{i}-{\bar{C}}_{m}\right)}^{2}}{N{\bar{C}}_{m}(1-{\bar{C}}_{m})}}$$

Furthermore, the mixing performance of the fluids in the channels was influenced not only by the mixing index, but also by the pressure drop, as the pressure drop had negative effects on the mixing performance. Therefore, we introduced the performance index (*PI*) to better characterize the mixing performance (as shown in Equation (7)), where Δ*p* was the pressure drop of the fluids between the inlet and the outlet. Thus, the micromixer with a larger *PI* had better mixing performance.

(7) $$PI=\frac{MI}{\Delta p}$$

The simulations were performed using commercial software called ANSYS Fluent 2023. The SIMPLE (Semi-Implicit Method for Pressure-Linked Equations) algorithm was employed for the pressure–velocity coupling. Second-order upwind schemes were applied for both the momentum and species transport equations to minimize numerical diffusion. The pressure was discretized using a second-order scheme. All simulations were run in steady state. The solution was considered converged when the scaled residuals of all equations fell below 10^−6^. As all simulations were steady-state, the setting of time steps was not applicable. The computational domain was discretized using tetrahedral elements with a refined boundary-layer mesh near all solid walls. The mesh was refined in the near-wall regions to ensure that the dimensionless wall distance y^+^ < 1 across all cases.

### 2.4. Grid Independence Verification and Validation of Validity

**Figure 2:**
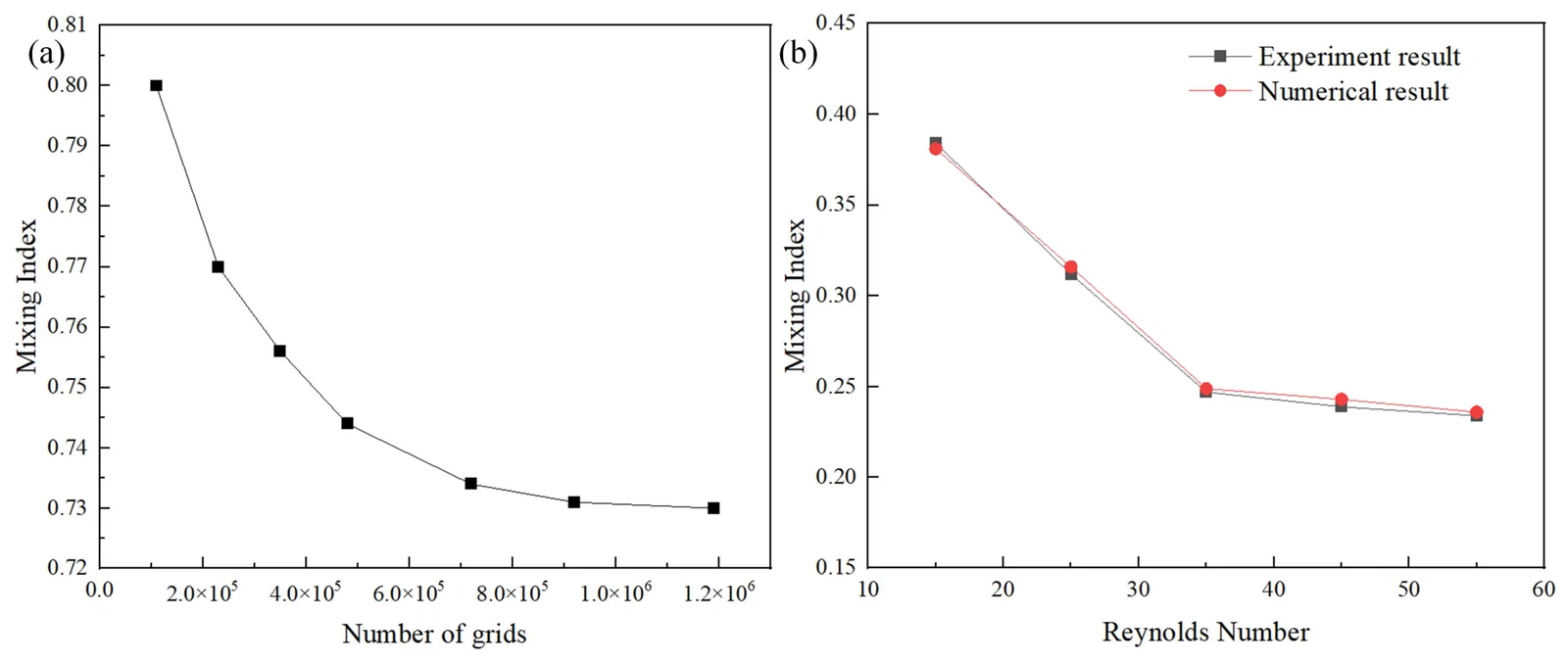
(**a**) Relationship between the number of grids and the mixing index. (**b**) Relationship between the Reynolds number and the mixing index of the experiment result and the numerical result.

The grid independence verification was a necessary step to avoid the deviation of the simulation results due to improper mesh settings and ensure the rationality and accuracy of the numerical simulation. In this work, we designed seven grid systems of 1.1 × 10^5^, 2.3 × 10^5^, 3.5 × 10^5^, 4.8 × 10^5^, 7.2 × 10^5^, 9.2 × 10^5^ and 1.19 × 10^6^ grids, respectively, for numerical simulations in which the Reynolds number was 50 (see Figure 2a). It can be observed that the mixing index difference between 9.2 × 10^5^ and 1.19 × 10^6^ grids was 0.21%, which means that the grid system of 9.2 × 10^5^ grids was good enough to keep the accuracy and was thus selected. Moreover, the same grid independence verification was carried out for other models’ simulation to maintain the accuracy of the numerical results. Figure 2b compares the experiment results [34] and the numerical results for different Reynolds numbers. In the numerical simulation in this paper, the geometry and boundary conditions of the channel were designed to match those in the reference. Although the reference used the D_I_/I_max_ to describe mixing efficiency rather than the MI, we could still obtain the MI from the D_I_/I_max_ through a calculation. As shown in the figure, there are negligible variations in the results, and the maximum change in the mixing index was only 1.67% when the Reynolds number was 45. These results demonstrated that the selected boundary conditions, mesh resolution, and numerical simulation methodology were reliable and sufficiently accurate for the present study.

## 3. Results and Discussion

### 3.1. Effects of Type of Variable Section Unit on Mixing

**Figure 3:**
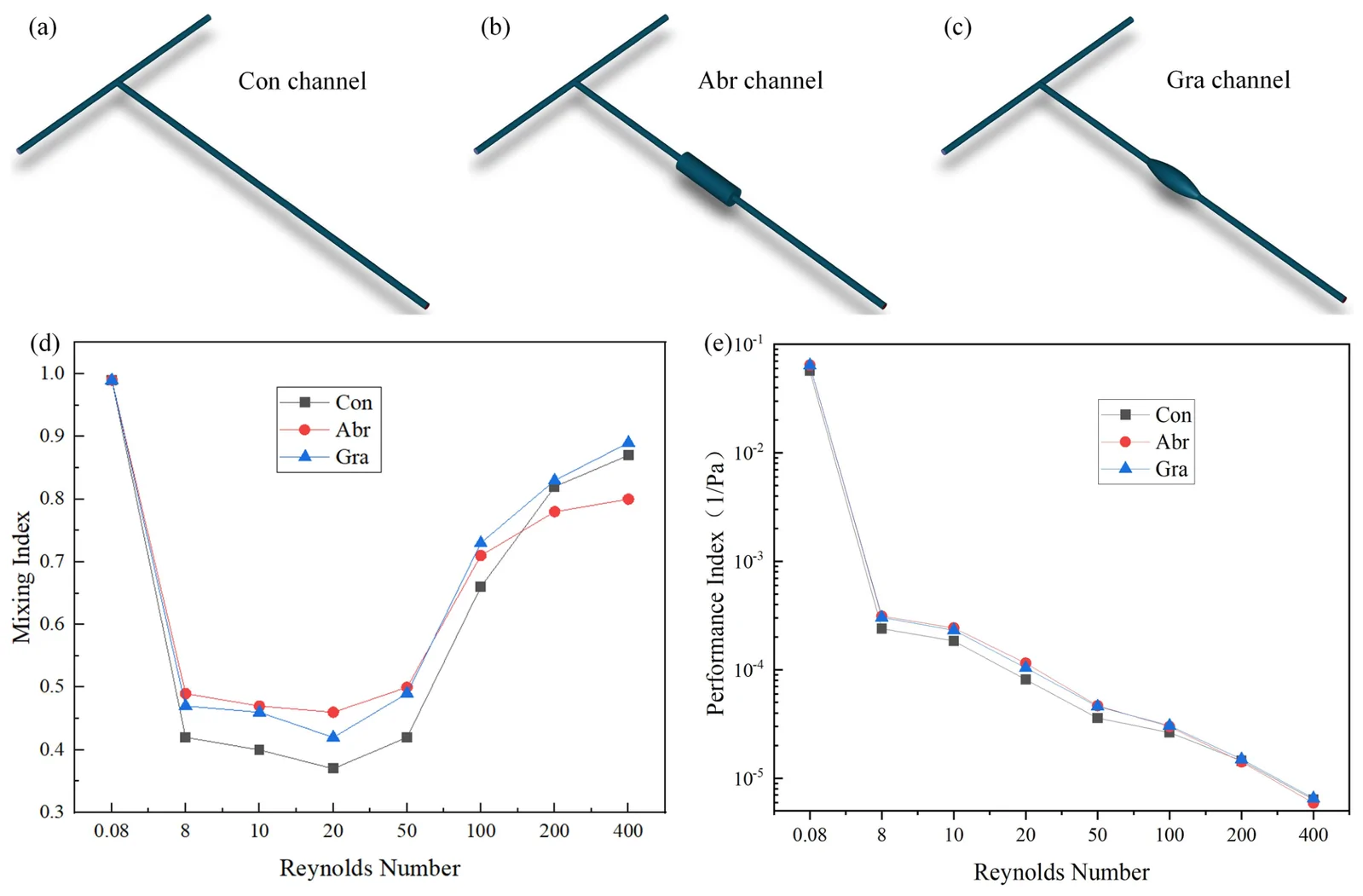
(**a**–**c**) Geometric models of the channels with variable section types of Con, Abr, and Gra respectively; (**d**) relationship between type of variable section unit and Reynolds number and mixing index; (**e**) relationship between type of variable section unit and Reynolds number and performance index.

The mixing index of the fluids in the channels was strongly influenced by the type of variable section unit. As shown in Figure 3a–c, we have compared the channels with constant section (referred to as Con channel), abruptly changing section (referred to as Abr channel), and gradually changing section (referred to as Gra channel). Different Reynolds numbers correspond to different residence times and lateral diffusion times of the fluids within the channel. Therefore, both the type of variable section unit and the Reynolds number can significantly affect the mixing process. The effects of types of variable section units and Reynolds numbers on mixing are shown in Figure 3d,e, in which Reynolds numbers are in the range of 0.08–400.

The fluids were almost completely mixed at a Reynolds number of 0.08 in all three channels, and the mixing index decreased as the Reynolds number increased from 0.08 to 20 (see Figure 3d). However, the mixing index increased with a further increase in the Reynolds number from 20 to 400. This behavior can be attributed to the transition from diffusion-dominated to convection-dominated mixing at higher Reynolds numbers. The variable section units enhanced convective transport, resulting in higher mixing indices for the Abr and Gra channels than for the Con channel. Figure 3e shows that the performance index decreased with increasing Reynolds number for all three channels. This decrease can be attributed to the increased pressure drop at higher Reynolds numbers, which led to a reduction in the overall performance index.

**Figure 4:**
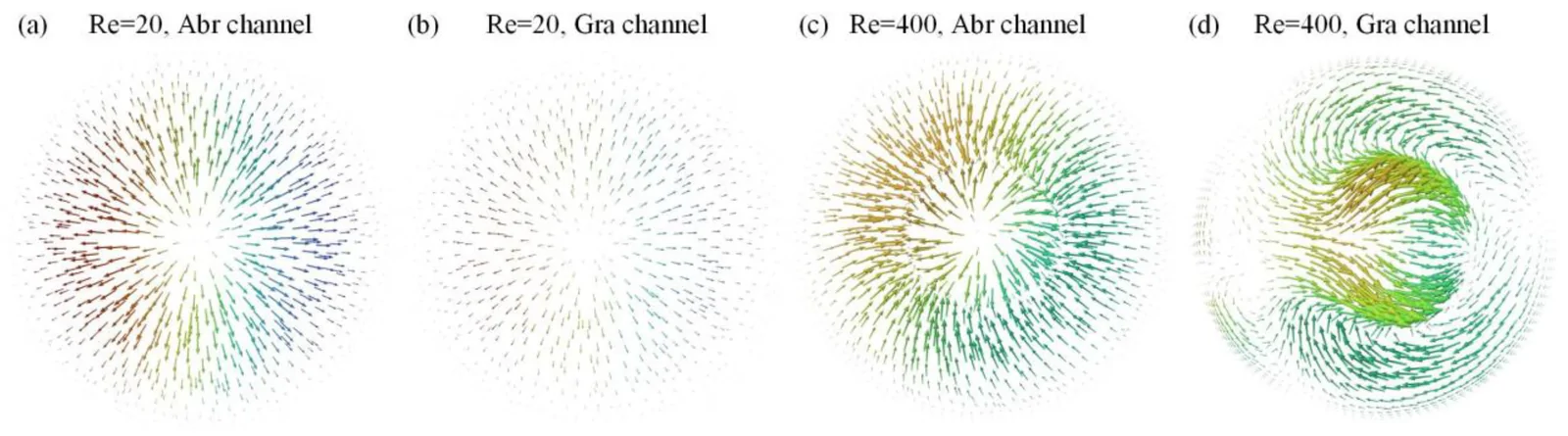
(**a**,**b**) Vector plots of salt water mass fraction at section A of the Abr channel and Gra channel at a Reynolds number of 20; (**c**,**d**) vector plots of salt water mass fraction at section A of the Abr channel and Gra channel at a Reynolds number of 400.

Figure 4a and Figure 4b show the vector plots of the salt water mass fraction at section A of the Abr and Gra channels, respectively, at a Reynolds number of 20. As shown in the figures, the vectors in the Abr channel were denser than those in the Gra channel at Re = 20, indicating that the Abr channel exhibited a higher mixing index than the Gra channel at this Reynolds number. However, at higher Reynolds numbers, the vector distribution at section A of the Gra channel became more complex than that of the Abr channel. The larger contact area between the two fluids enhanced convective transport, promoted vortex formation, and consequently improved the mixing performance (see Figure 4c,d). Therefore, the Gra configuration was selected as the type of variable section unit because it provided the best mixing performance.

### 3.2. Effects of the Position of the Variable Section Unit on Mixing

**Figure 5:**
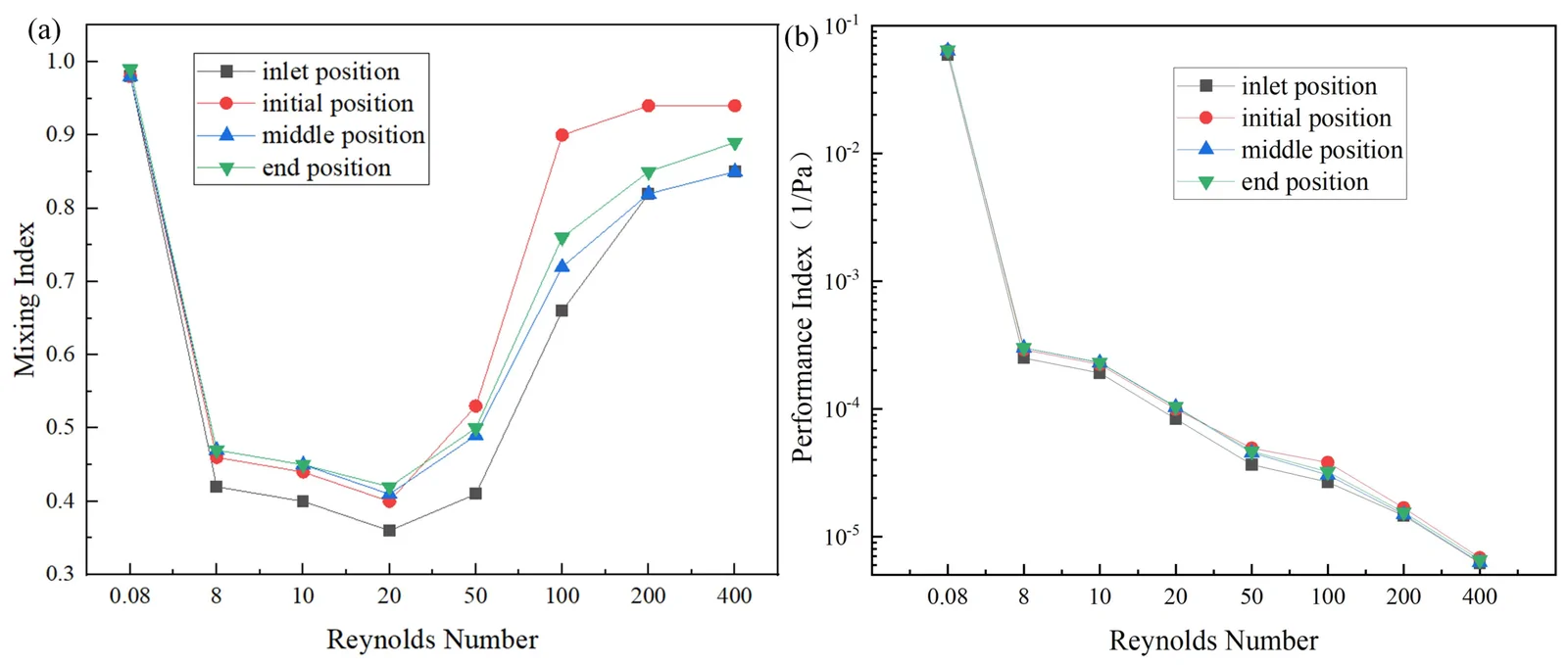
(**a**) Relationship between the position of the variable section unit and Reynolds number and mixing index; (**b**) relationship between the position of the variable section unit and Reynolds number and performance index.

Different positions of the variable section units along the channel can produce different flow patterns and consequently affect the mixing process. In this study, the positions of the variable section units include the middle position of the inlet region (referred to as the inlet position channel), the initial position of the mixing region (referred to as the initial position channel), the middle position of the mixing region (referred to as the middle position channel), and the end position of the mixing region (referred to as the end position channel). The effects of the variable section unit position and Reynolds number on the mixing performance are shown in Figure 5, with the Reynolds number ranging from 0.08 to 400.

When the Reynolds number ranged from 0.08 to 20, the end position channel exhibited the highest mixing index, whereas the inlet position channel exhibited the lowest among the four configurations (Figure 5a). This can be attributed to the dominance of intermolecular diffusion at low Reynolds numbers. In the end position channel, the fluids stayed for a longest time and made best use of the intermolecular diffusion effects for mixing, which led to a best mixing index.

**Figure 6:**
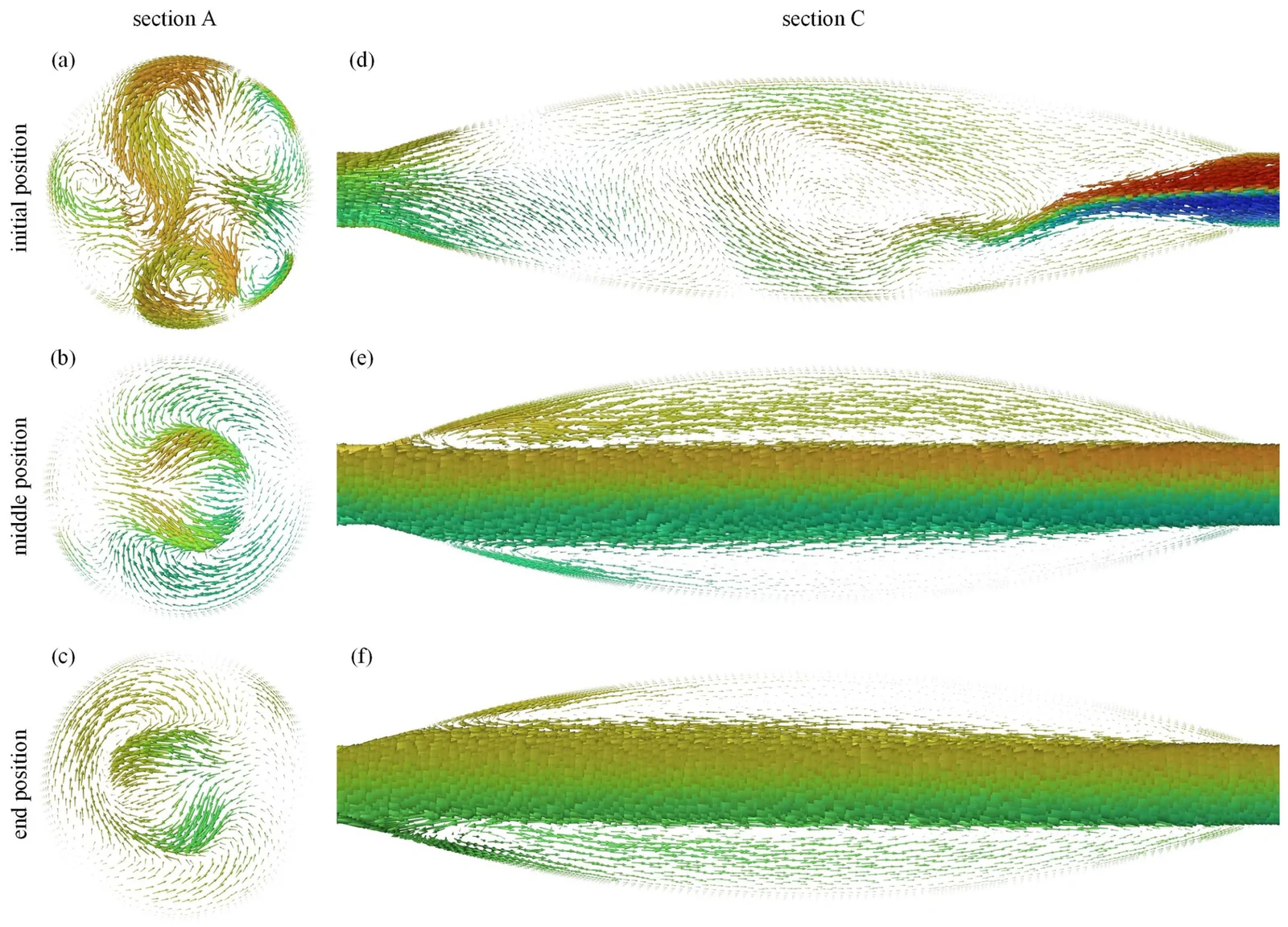
(**a**–**c**) Vector plots of salt water mass fraction at section A of the initial position channel, the middle position channel, and the end position channel, respectively, at a Reynolds number of 400; (**d**–**f**) vector plots of salt water mass fraction at section C of the initial position channel, the middle position channel, and the end position channel, respectively, at a Reynolds number of 400.

However, when the Reynolds number ranged from 50 to 400, the initial position channel exhibited the highest mixing index among the four configurations. Figure 6a–c show that the fluids in the initial position channel were very complex with large numbers of intense vortex phenomena in all directions at section A, but in the middle position channel and end position channel, the vortex phenomena were less intense. As shown in Figure 6d, when the two fluids met in the mixing region, the flow velocity and direction had not yet stabilized due to the greater value Reynolds number. The variable section at the initial position could help change the flow velocity and direction again. Due to the differences between the properties of the two fluids, the flow direction of the fluid may have continuously shifted to the edge of the channel, which led to a larger scale vortex and promoted the mixing process.

**Figure 7:**
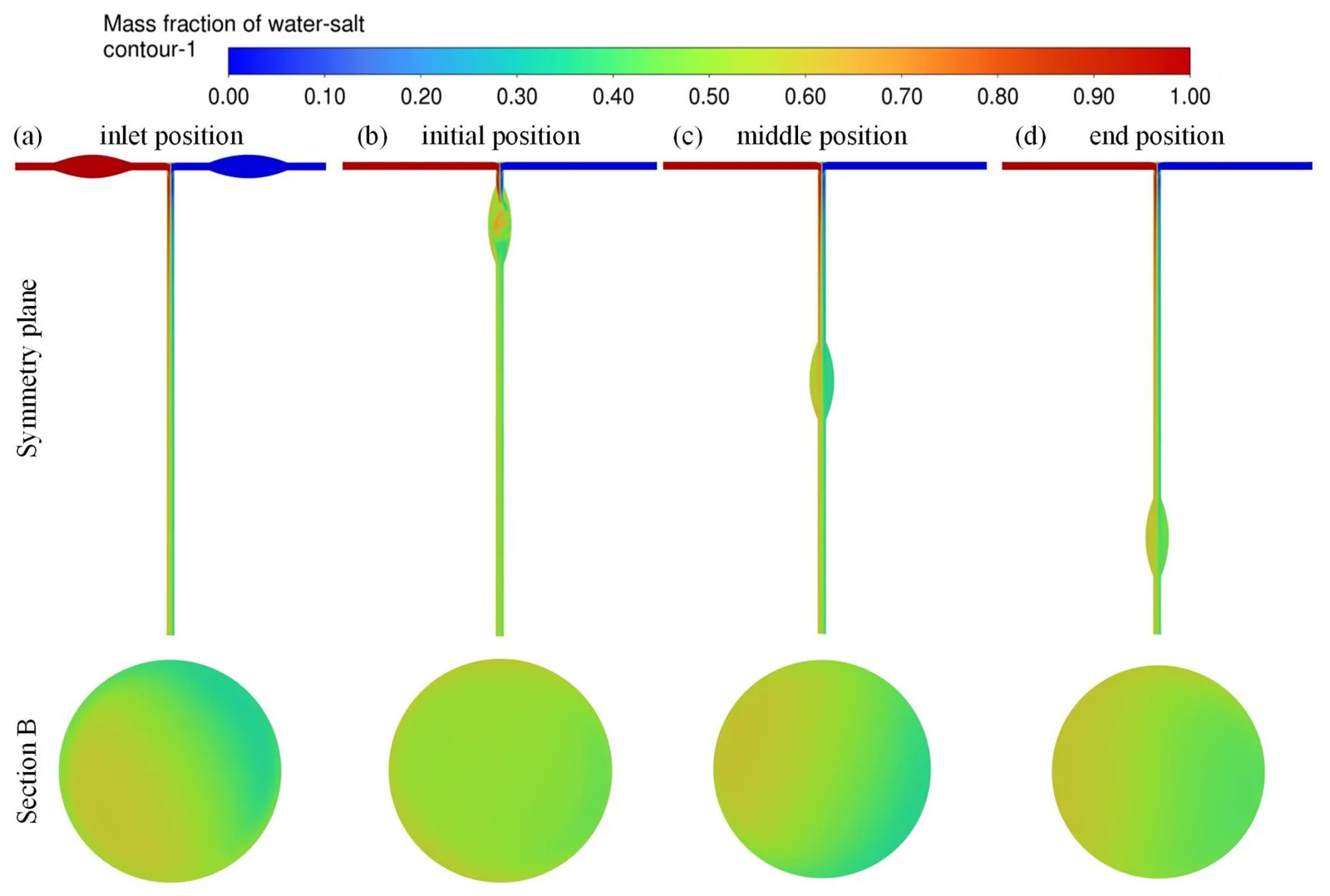
(**a**–**d**) Cloud plots of salt water mass fraction at the symmetry plane and section B of the inlet position channel, the initial position channel, the middle position channel, and the end position channel, respectively, at a Reynolds number of 400.

Figure 7 shows the cloud plots of the salt water mass fraction in the channels with different positions of variable section units during the mixing process at a Reynolds number of 400. As a result, when the variable section unit was set to the initial position of the mixing region, the liquids were well mixed. Figure 5b also shows that the variable section unit in the initial position had the best performance index among the four positions.

### 3.3. Effects of the Number of Variable Section Units on Mixing

The formation of vortices at the variable section units enhanced convective transport within the fluids. Therefore, increasing the number of variable section units (referred to as N) was expected to further enhance mixing efficiency. In this paper, the total length of the mixing region was fixed with a value of 12 mm. The mixing region could be divided into three parts: a merging part with a length of 0.5 mm, a variable section part with a length of 10 mm, and an outlet part with a length of 1.5 mm. In the variable section part, the variable section units were configured according to the structural parameters shown in Table 2. For example, when the N was 6, the length of each variable section unit was 1.33 mm, and the distance between the neighboring units was 0.4 mm.

**Figure 8:**
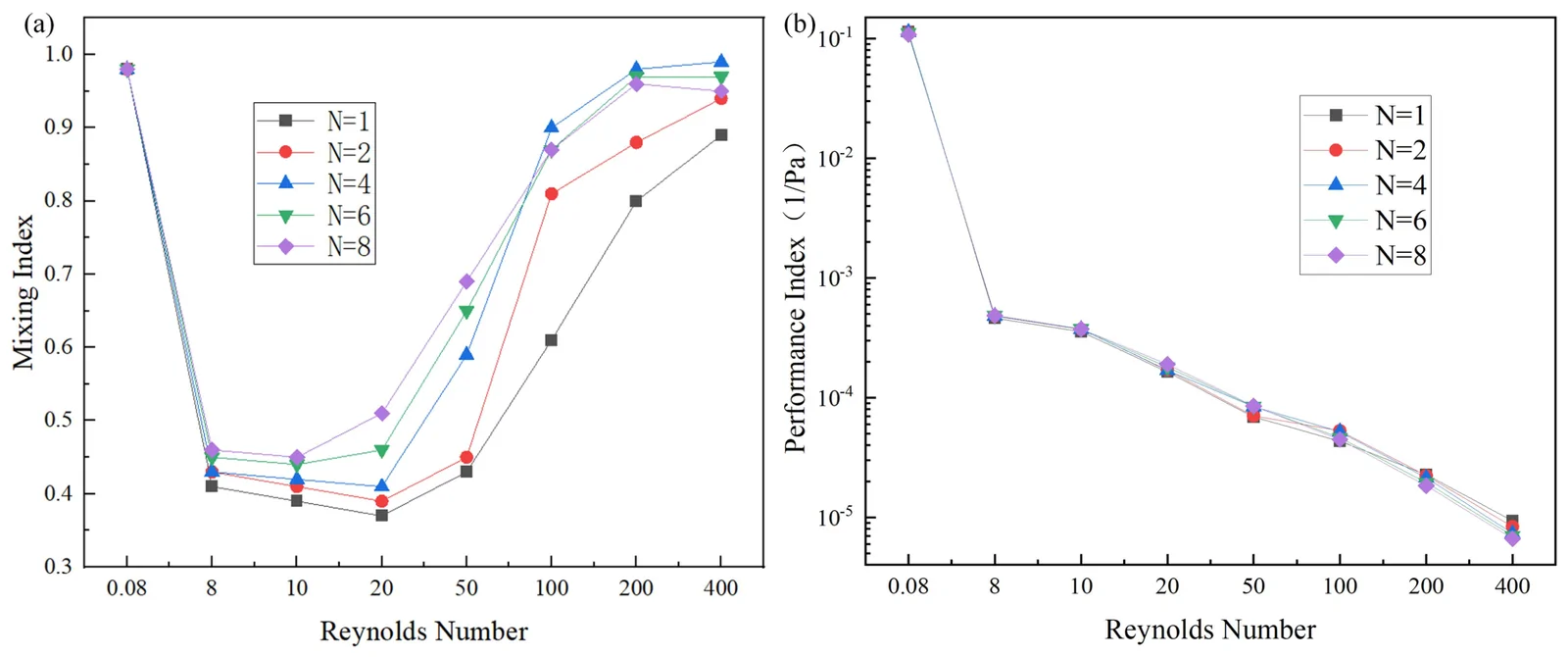
(**a**) Relationship between number of the variable section units and Reynolds number and mixing index; (**b**) relationship between number of the variable section units and Reynolds number and performance index.

Figure 8 shows the effects of N on mixing, in which the N values were 1, 2, 4, 6, and 8, respectively. As shown in Figure 8a, the mixing index decreased as the Reynolds number increased from 0.08 to 20. However, when the Reynolds number increased from 20 to 400, the increasingly complex flow structures induced by the variable section units enhanced convective transport between the fluids, resulting in a rapid increase in the mixing index. When the Reynolds number reached 100 or higher, the channel with an N value of 4 exhibited the highest mixing index among the investigated configurations.

**Figure 9:**
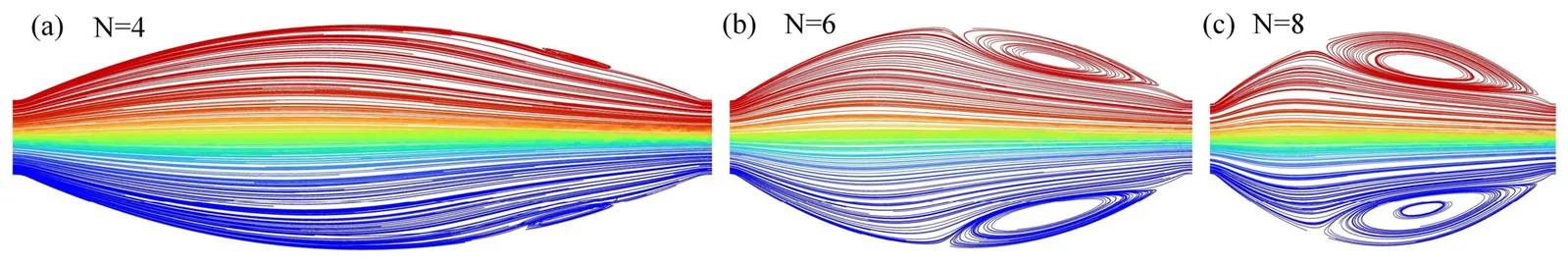
(**a**–**c**) Streamline diagrams of the fluids at section C of the channels with numbers of 4, 6, and 8, respectively, and a Reynolds number of 20.

Figure 9 shows the streamlines of the fluids in different channels with numbers of 4, 6 and 8, respectively, and a Reynolds number of 20. As can be seen from the figure, vortices occurred in the channel with an N value of 4, and as the N increased from 4 to 8, both the range and intensity of the vortices increased, which enlarged the contact area and enhanced the mixing efficiency.

**Figure 10:**
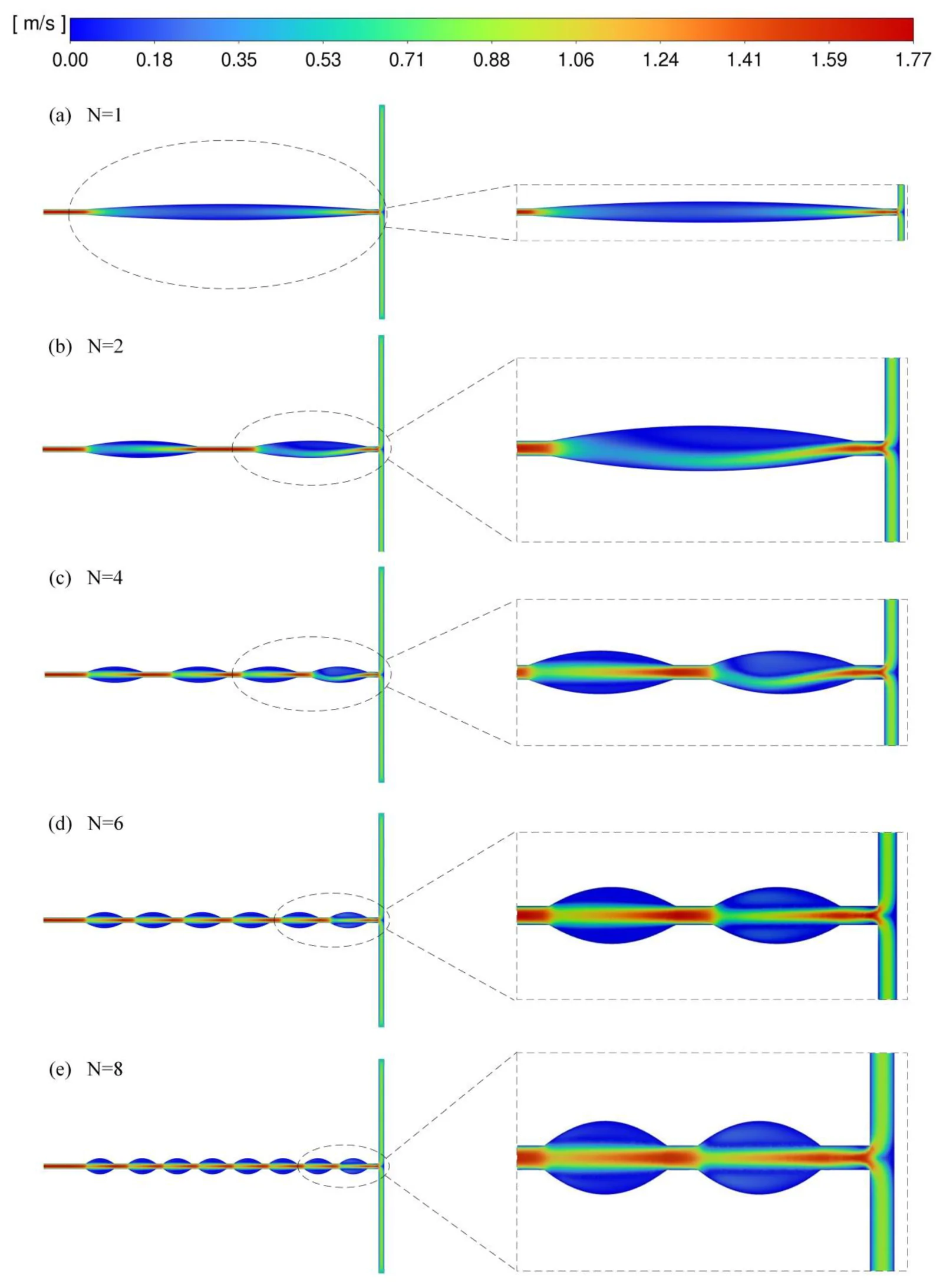
(**a**–**e**) Cloud plots and enlarged cloud plots of fluid velocity in the symmetrical plane of the channels with numbers of 1, 2, 4, 6, and 8, respectively, and a Reynolds number of 100.

Figure 10 shows the fluid velocity contours in channels with numbers of 1, 2, 4, 6, and 8 and a Reynolds number of 100. When the N was 1, the variable section unit was relatively long, providing sufficient space and residence time for the flow velocity and direction to stabilize. As a result, the disturbance induced by the variable section unit was relatively weak, and the fluids could not fully utilize the enhanced convective transport for mixing. As N increased from 1 to 4, the variable section units generated more frequent disturbances in the flow field, resulting in more pronounced convective transport and an increase in the mixing index. However, when N exceeded 4, the length of each unit became too short, which led the fluid to converge in the middle of the channel, as it was unable to flow towards the edges during the mixing process. This reduced the range and intensity of vortices, thereby reducing the mixing index. Therefore, the channel with the N of 4 exhibited the highest mixing index. In addition, the channel with the N of 4 achieved the highest performance index among all configurations at a Reynolds number of 100. Therefore, the number of variable section units of the channel was considered to be 4.

### 3.4. Effects of the Ratio of Variable Section Units on Mixing

**Figure 11:**
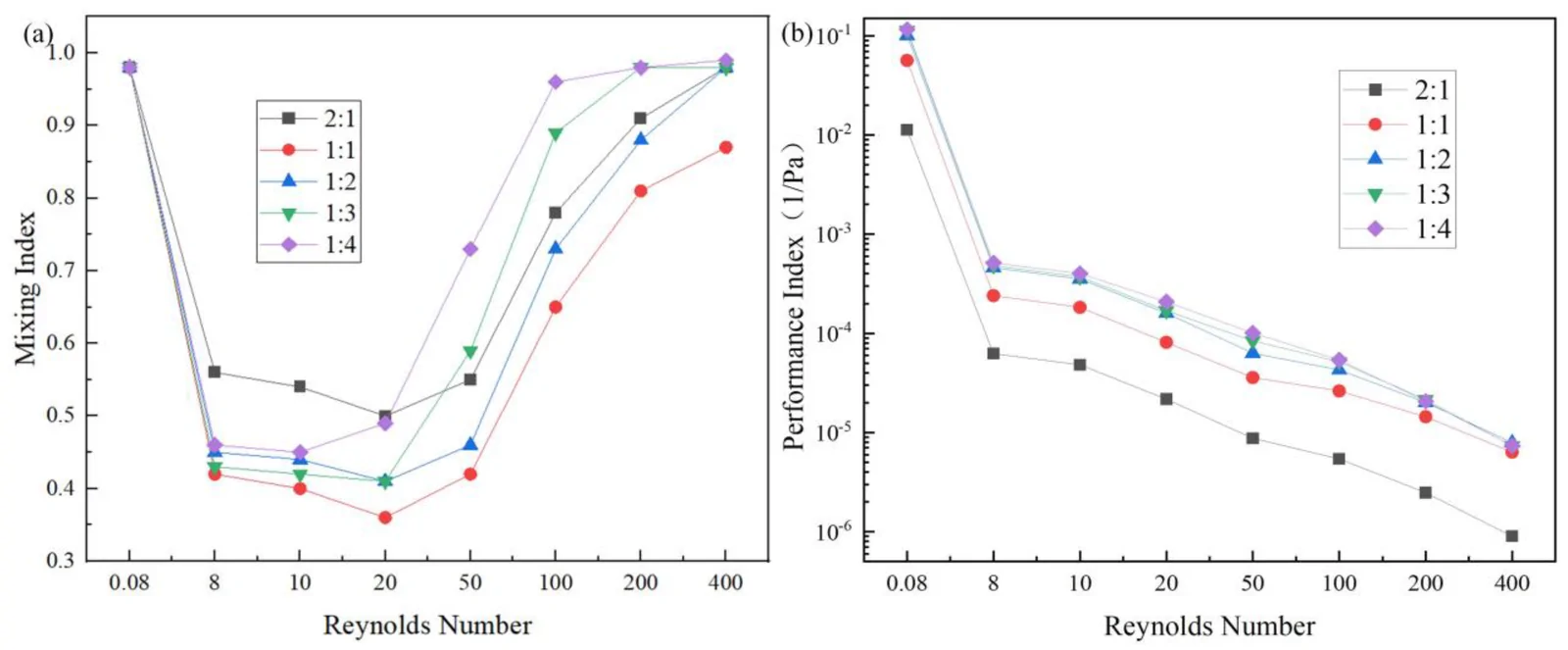
(**a**) Relationship between ratio and Reynolds number and mixing index; (**b**) relationship between ratio and Reynolds number and performance index.

The effects of the contraction ratio and expansion ratio of the variable section units (referred to as ratio) on mixing were researched. The ratios were 2:1, 1:1, 1:2, 1:3, and 1:4, and the results are shown in Figure 11a. When the Reynolds number ranged from 0.08 to 20, the channel with a ratio of 2:1 exhibited the highest mixing index among all the investigated configurations. This is because the diameter of the channel was small, and the intermolecular diffusion dominated the mixing process and contributed to a higher mixing index. However, the smaller channel diameter also resulted in a larger pressure drop, leading to the lowest performance index among the investigated configurations (see Figure 11b).

**Figure 12:**
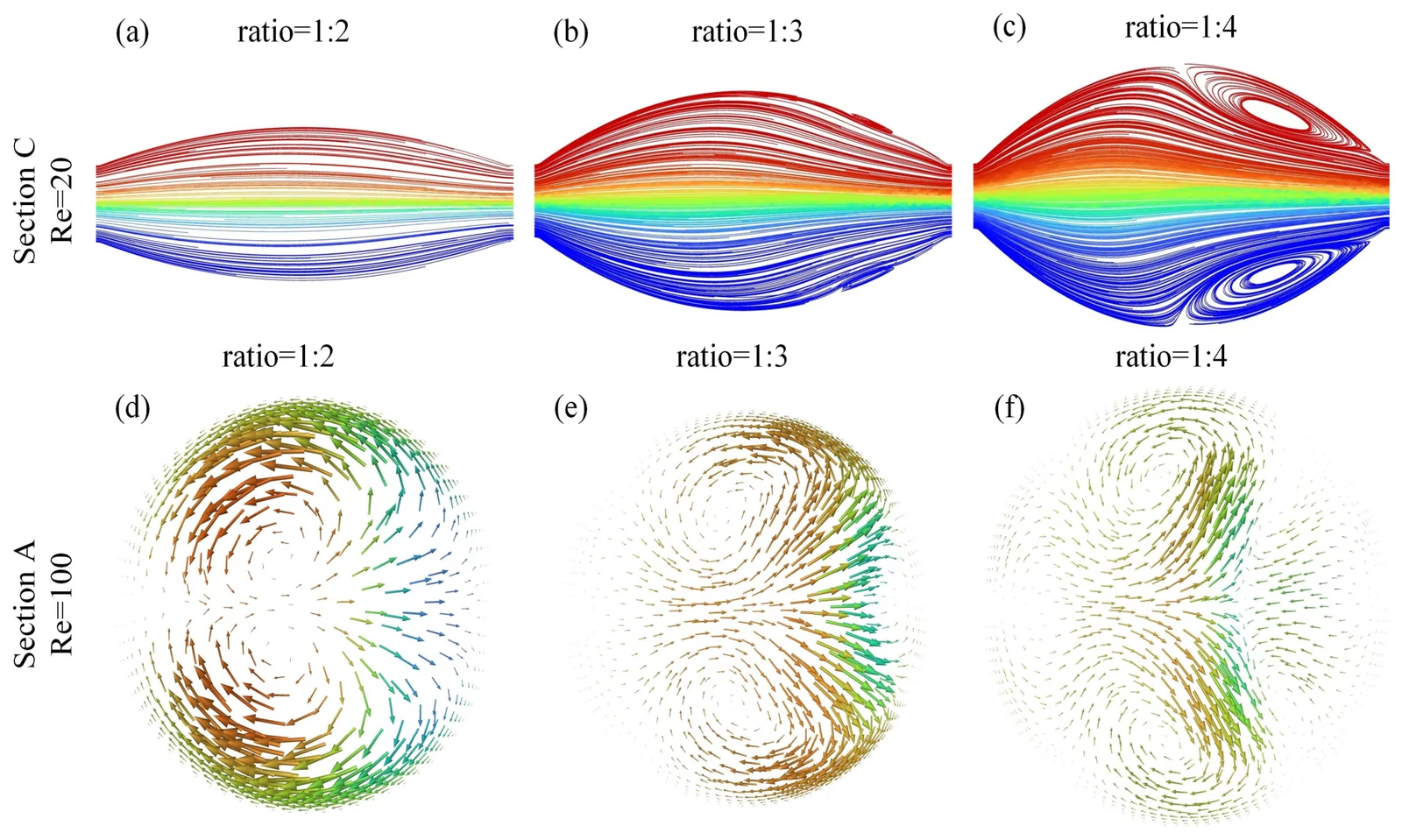
(**a**–**c**) Streamline diagrams of fluids at section C of the channels with ratios of 1:2, 1:3, and 1:4, respectively, at a Reynolds number of 20; (**d**–**f**) vector plots of salt water mass fraction at section A of the channels with ratios of 1:2, 1:3, and 1:4, respectively, at a Reynolds number of 100.

Figure 11a shows that the mixing index increased when the ratio increased from 1:1 to 1:4 as the diameter of the channel section became larger. As shown in Figure 12a–c, the variation in the channel section was relatively gradual at a ratio of 1:2, resulting in a relatively low mixing index. When the ratio increased to 1:3, small-scale vortices could be found. At a ratio of 1:4, larger-scale vortices were generated, resulting in more pronounced convective transport. These results indicate that greater changes in the channel section could induce stronger flow disturbances and consequently enhanced the mixing performance. As shown in Figure 12d,e, at a Reynolds number of 100, increasing the ratio resulted in the formation of more vortices, which increased the interfacial area between the two fluids and enhanced convective transport. Consequently, the mixing index increased. The channel with a ratio of 1:4 achieved the highest mixing index of 0.96, indicating that the fluids were nearly completely mixed.

**Figure 13:**
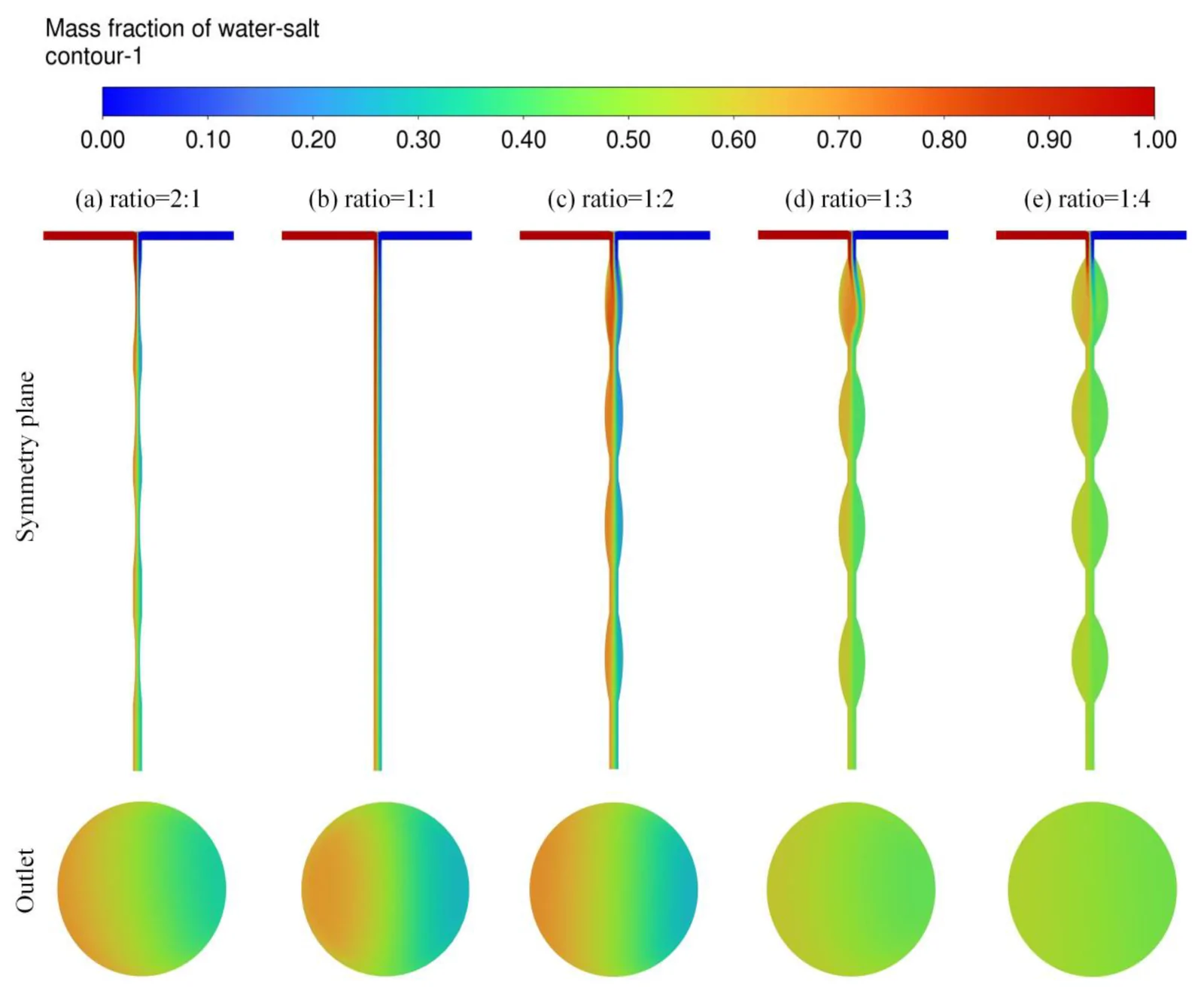
(**a**–**e**) Cloud plots of salt water mass fraction at the symmetry plane and section B of the channels with ratios of 2:1, 1:1, 1:2, 1:3, and 1:4, respectively, at a Reynolds number of 100.

Figure 13 shows the saltwater mass fraction contours during the mixing process at a Reynolds number of 100. The results further demonstrate that the channel with a ratio of 1:4 exhibited the best mixing performance. As shown in Figure 11b, the channel with a ratio of 1:4 also achieved the highest performance index. This can be attributed to the larger channel diameter associated with a higher ratio, which resulted in a lower pressure drop and consequently improved the performance index. Therefore, the channel with the ratio of 1:4 was selected.

We have built a theoretical framework for making passive micromixers with high mixing efficiency. Future work could be focused on experimental work in which nanomaterials [35] and advanced synthesis technology [36,37] can be used to make passive micromixers.

## 4. Conclusions

In this study, we have proposed a novel passive micromixer with variable section units through changing the geometric parameters including the type, position, number, and ratio of variable section units. Numerical analysis and parametric comparison of the effect of geometric parameters on the mixing performance were carried out. The selected variable section unit was Gra-type, as it had the best mixing index and performance index at higher Reynold numbers. The selected position of variable section unit was the initial position, because the variable section in the initial position could help to change the flow velocity and direction, which continuously shifted to the edge of the channel, leading to a larger scale vortex and promoting the mixing process. Creating more variable section units could improve mixing efficiency as more vortices are created. However, this improvement no longer applies when the value of N is larger than 4, as the length of each unit becomes shorter. This shorter length makes the fluid focus in the middle of the channel and unable to flow towards the edges during the mixing process, reducing the range and intensity of vortices. The selected ratio of the variable section unit was 1:4, as more vortices occurred, which greatly increased the contact area between the fluids and improved the mixing index. Furthermore, its performance index was the best, as the channel with the larger ratio caused a smaller pressure drop. In this way, the mixing efficiency of the selected micromixer was greatly improved, and the mixing index was able to reach up to more than 0.96. To conclude, our work theoretically created passive mixers with good mixing efficiency.

## Figures and Tables

**Table 1:** Velocity and Reynolds number.

Velocity (m/s)	0.0004	0.04	0.05	0.1	0.25	0.5	1	2
Re	0.08	8	10	20	50	100	200	400

**Table 2:** Structural parameters for different variable section unit numbers.

N	Structural Parameters
0	10 mm
1	8 mm–2 mm
2	4 mm–2 mm–4 mm
4	2 mm–0.5 mm–2 mm–0.5 mm–2 mm–1 mm–2 mm
6	1.33 mm–0.4 mm–1.33 mm–0.4 mm–1.33 mm–0.4 mm–1.33 mm–0.4 mm–1.33 mm–0.4 mm–1.33 mm
8	1 mm–0.25 mm–1 mm–0.25 mm–1 mm–0.25 mm–1 mm–0.25 mm–1 mm–0.25 mm–1 mm–0.25 mm–1 mm–0.5 mm–1 mm

## Data Availability

The original contributions presented in this study are included in the article. Further inquiries can be directed to the corresponding authors.
